# The Missouri Valley Veterinary Association

**Published:** 1900-12

**Authors:** B. F. Kaupp

**Affiliations:** Secretary pro tem.


### THE MISSOURI VALLEY VETERINARY ASSOCIATION.

THE twenty-fifth regular meeting was held in Kansas City, October 29, 1900. The following veterinarians were present: Drs. W. R. Andress, R. Blanch, S. E. Bennett, F. F. Brown, R. H. Carswell, W. J. Fretz, John Forbes, B. F. Kaupp, R. C. Moore, N. B. Smith, S. Stewart, D. C. Hannawalt, James Wilson, J. B Wright, E. Lee, and about fifty veterinary students.

From 2 to 5 P.M. a very instructive surgical clinic was held in the operating-rooms of the Kansas City Veterinary College, which included a variety of recent operations.

At 7.30 P.M. the meeting was called to order by the President, Dr. John Forbes, of St. Joseph. After roll-call and reading of minutes of previous meeting, Dr. S. Stewart, of Kansas City, Chairman of the Legislative Committee, gave an account of the work being done by that committee and by the Committee of the Missouri Veterinary Medical Association toward securing legislation to regulate the practice of veterinary medicine in the State of Missouri. The report was accepted and the committee continued.

The following programme was presented: Dr. S. E. Bennett, "Age of the Horse as Revealed by the Teeth." Dr. S. Stewart, "Report of the Thirty-seventh Annual Meeting of the American Veterinary Medical Association." Dr. B. F. Kaupp, "A Report of the Ninth Annual Meeting of the Missouri Veterinary Medical Association." Drs. E. J. Netherton and C. E. Steele, "Reports of Cases." A generous and spirited discussion was elicited by the papers.

The next meeting will be held in St. Joseph, in December, 1900.

B. F. KAUPP, D.V.S.,
Secretary pro tem.

